# Assessing vocal changes through spectral analyses of vocalizations in a cerebellar-specific dystonia mouse model

**DOI:** 10.3389/dyst.2026.15530

**Published:** 2026-03-26

**Authors:** Austin L. Fitzgerald, Jonathan A. Coello, Alyssa M. Lyon, Breanne L. Dao, Meike E. van der Heijden

**Affiliations:** 1 Fralin Biomedical Research Institute at Virginia Tech Carilion School of Medicine, Roanoke, VA, United States,; 2 Graduate Program in Translational Biology, Medicine, and Health, Virginia Polytechnic Institute and State University, Blacksburg, VA, United States,; 3 Center for Neurobiology Research, Fralin Biomedical Research Institute, Roanoke, VA, United States,; 4 School of Neuroscience, Virginia Polytechnic Institute and State University, Blacksburg, VA, United States,; 5 Department of Pediatrics, Virginia Tech Carilion School of Medicine, Roanoke, VA, United States

**Keywords:** cerebellar dysfunction, early-onset dystonia, mouse model, translational neuroscience, ultrasonicvocalizations

## Abstract

Vocal impairments are a debilitating but understudied feature of several dystonias, including generalized and early-onset genetic forms. Despite growing recognition that cerebellar dysfunction contributes to dystonic pathophysiology, the circuit mechanisms underlying vocal-motor abnormalities remain poorly understood, and effective treatments remain limited, in part due to the lack of a preclinical model that captures specific vocal features. Our experiment evaluates ultrasonic vocalizations (USVs) in *Ptf1a*^*Cre/+*^*;Vglut2*^*fl/fl*^ mice, a cerebellum-specific generalized dystonia model, to assess cerebellar contributions to phonation and explore translational relevance for vocal features. At postnatal day 9, dystonic mice demonstrated statistically significant reductions in total USV count, relative count of complex calls, and key spectral parameters—especially frequency modulation and power—mirroring general phonatory abnormalities seen in dystonia. Cluster analyses further revealed impaired vocal burst initiation, suggesting disrupted cerebellar coordination of temporal vocal-motor output. These findings support the model’s construct and face validity for studying cerebellar mechanisms of vocal impairment. By identifying quantifiable acoustic disruptions, our study establishes a foundational platform for future circuit-targeted investigations of vocal-motor dysfunction in dystonia.

## Introduction

Vocal impairments in dystonia are increasingly understood within a broader network framework involving disrupted motor planning, abnormal sensorimotor integration, and impaired inhibition. Neuroimaging has identified both structural and functional alterations in multiple areas involved in motor control, such as the basal ganglia, thalamus, sensorimotor cortex, supplementary motor area, and cerebellum. A recent meta-analysis compiling data from over 500 patients with dystonia affecting the voice showed consistent abnormalities in motor and sensorimotor regions of the brain, supporting the view that vocal symptoms arise from distributed dysfunction of the networks responsible for planning and coordination of voice production rather than isolated peripheral abnormalities [1].

Among the implicated brain regions, the cerebellum is receiving increasing attention in dystonia research. Traditionally associated with coordination, balance, and motor learning, the cerebellum is now better understood to play important roles in speech timing and fine motor control of the vocal apparatus. Functional imaging studies in patients with dystonia involving vocal symptoms have demonstrated cerebellar hyperactivation during speech tasks, indicating its involvement in vocal motor dysregulation [2]. These disruptions may contribute to symptoms like impaired pitch and intensity modulation. Human studies also show exaggerated pitch-shift reflexes and impaired auditory-motor adaptation, reflecting a hyper-reactive yet poorly calibrated vocal feedback loop [3–8].

It is well established that cerebellar dysfunction, such as in hereditary or acquired ataxias, can result in speech abnormalities like ataxic dysarthria, which is characterized by slurred, poorly coordinated articulation and irregular rhythm [9]. However, it remains unclear whether cerebellar circuit disruption alone can produce vocal-motor abnormalities more characteristic of dystonia. Addressing this gap is essential for clarifying cerebellar contributions to vocal impairments across dystonia syndromes. Our use of a targeted cerebellar dysfunction model enables investigation of whether specific circuit lesions within the cerebellum can yield vocal-motor abnormalities, advancing our understanding of cerebellar contributions to speech coordination beyond the established ataxic spectrum.

Vocal impairments are a clinically significant but under characterized feature of several dystonia syndromes, including generalized and inherited forms, and these abnormalities in voice production can substantially impact communication and quality of life. Despite this clinical burden, current treatments for vocal dystonia are temporary and results vary widely, with the neural mechanisms underlying vocal-motor dysfunction in dystonia remaining incompletely understood [10–13]. In some DYT1 (TOR1A) families, cranial or cervical symptoms have been documented in childhood and can precede limb involvement, although this remains much less common than limb-first presentations [14, 15]. In DYT6 (THAP1) dystonia, early laryngeal involvement is typical and often dominates the initial phenotype [16]. These phenotypic patterns suggest that the circuits governing voice production are particularly vulnerable in certain genetic forms of dystonia. Studying cerebellar-specific contributions to vocal control may offer insight into early disease progression and highlight therapeutic windows missed by limb-focused studies. The emergence of laryngeal symptoms during childhood in certain human genetic forms of dystonia lend merit to investigating cerebellar dysfunction during key neurodevelopmental periods.

Developing targeted treatments requires circuit-level models that capture key features of disordered phonation [17]. However, most existing rodent models of dystonia are primarily striatal or cortical and recapitulate generalized or limb motor dysfunction without a vocal phenotype [18, 19]. This gap has limited preclinical insights into the mechanisms underlying vocal impairments, especially those involving cerebellar circuits. However, mouse ultrasonic vocalizations (USVs) offer a promising solution.

While mice do not produce speech like people, their USVs are high-frequency, structured vocal signals that require precise coordination of laryngeal and respiratory muscles. These calls rely on intact cerebellar and auditory feedback loops, making them a surprisingly rich analog for vocal-motor function [20]. In other species, such as songbirds and humans, the cerebellum has been increasingly recognized as critical for learned vocal communication, including pitch control and timing [21, 22]. These findings suggest that cerebellar contributions to vocal learning and modulation may be evolutionarily conserved. While this relationship is less established in mice, newer evidence does implicate the cerebellum in behavioral production of speech in mice, and recent studies underscore the importance of cerebellar circuits in fine-tuning vocal output across species [23]. USVs can be quantitatively analyzed for call type, frequency, modulation, power, and duration—features that may parallel vocal impairments observed across dystonia syndromes, particularly in early-onset cases [24].

Our study uses a well-characterized cerebellum-specific mouse model of generalized dystonia (*Ptf1a*^*Cre/+*^*;Vglut2*^*fl/fl*^) to explore whether targeted circuit disruption can yield vocal-motor impairments relevant to dystonia syndromes. This model replicates dystonic movements that improve with cerebellar-targeted DBS and meets key standards for animal model validity [1]: face validity—visible muscle contractions similar to those in patients [2]; construct validity—abnormal cerebellar function consistent with human and rodent dystonia; and [3] predictive validity—postural and limb symptoms respond to cerebellar DBS, paralleling patient outcomes [25, 26]. This study focused on postnatal day 9 (P9) mice due to this time point being a developmental stage where cerebellar circuits are actively maturing and USVs are reliably produced [27, 28].

In this manuscript, we set out to investigate whether mice with cerebellar dysfunction-related generalized dystonia exhibit USV abnormalities relevant to early-onset and generalized dystonias featuring voice impairments. Rather than modeling a single focal subtype, this work establishes a circuit-level platform for probing how cerebellar dysfunction affects vocal-motor control, with broader translational relevance for disorders of phonation.

## Materials and methods

### Animal models and ethical compliance

All procedures were approved by the Virginia Tech Institutional Animal Care and Use Committee (IACUC Protocol #23–181) and adhered to NIH guidelines for the care and use of laboratory animals. Experiments were performed on C57BL/6J mice, with the dystonic group comprised of *Ptf1a*^*Cre/+*^*;Vglut2*^*fl/fl*^ mice (n = 23; 18 females, 5 males). This genetic mouse model represents a conditional knockout where all climbing fiber signals to Purkinje cells from the inferior olive are eliminated, resulting in complete absence of glutamatergic neurotransmission and subsequent cerebellar-specific inability to effectively “turn off” cerebellar nuclei signals, producing an extreme dystonic phenotype [29]. Littermate *Vglut2*^*fl/fl*^ mice served as healthy controls (n = 27; 11 females, 16 males). Both sexes were used, and all recordings were conducted at P9, a developmental timepoint when pups vocalize reliably following maternal separation and cerebellar circuits are undergoing active refinement [27, 28]. This developmental window is particularly relevant given the early onset of vocal symptoms in some genetic dystonias (e.g., THAP1), allowing investigation of how cerebellar circuit dysfunction may affect phonation during critical neurodevelopmental stages.

### Experimental design and recording setup

Mouse pups (n = 50 from 9 litters) were separated from the dam and immediately and individually placed in a sound-isolated Metris SmartChamber for 120 s of free vocalization. USVs were recorded using a Metris Gold Foil Electrostatic Transducer and digitized at a sampling rate of 250 kHz. Spectrograms were analyzed using Metris Sonotrack software (v1.4.7, Metris B.V., Netherlands).

This USV experiment was designed to assess cerebellar involvement in early vocal production by comparing vocalization quantity, acoustic structure, and temporal dynamics between dystonic and control pups. Nine animals emitting 10 or fewer calls during the 120 s recording period were excluded according to a pre-established criterion to ensure statistical reliability.

All mice from available litters were recorded, and vocalizations were recorded in a consistent environment. Investigators were blinded to genotype during data collection.

### USV parameter definitions and classification

**FIGURE 1:**
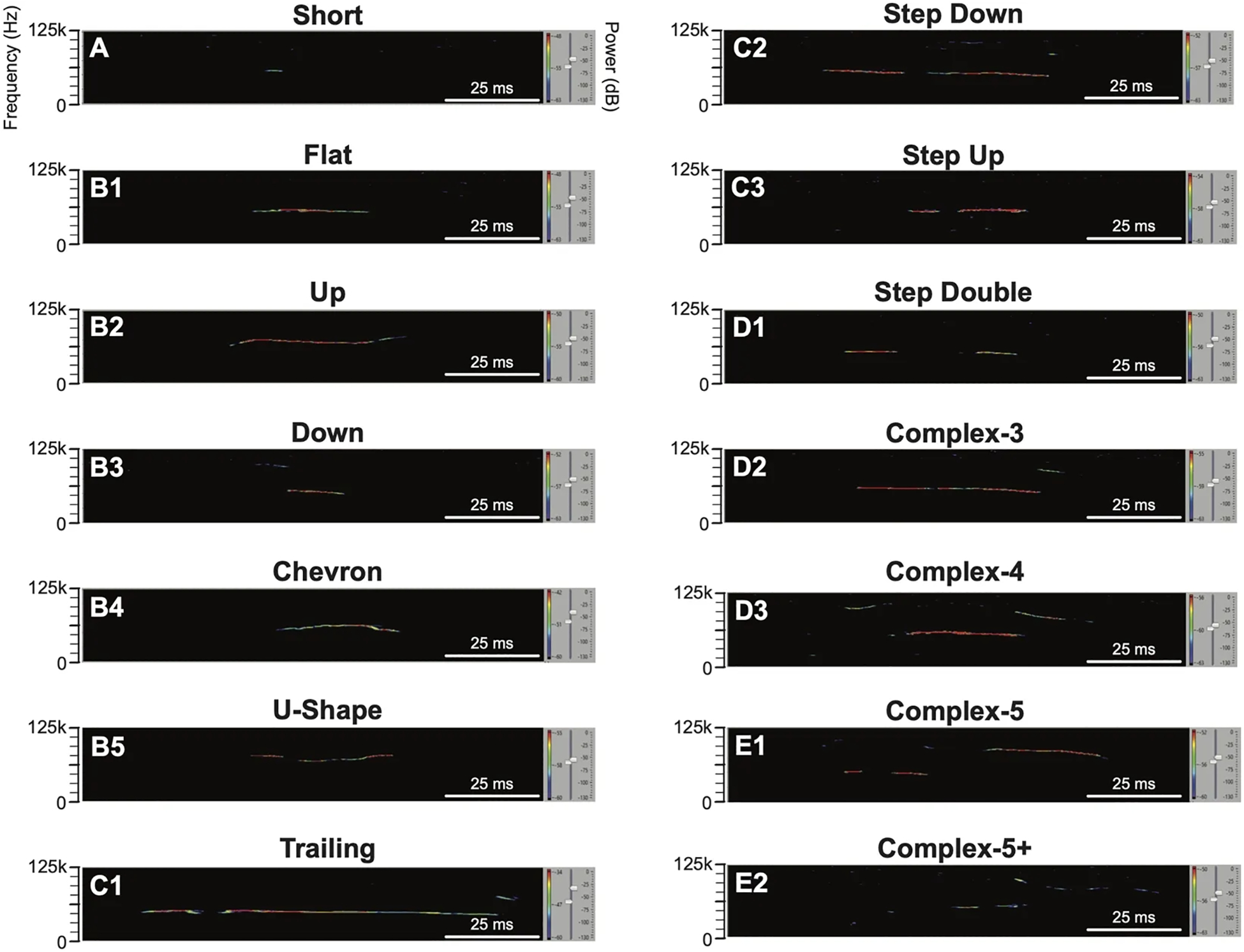
Example spectrograms of categorized USV call types recorded from P9 mouse pups. Each panel shows an example USV spectrogram annotated by the Metris Sonotrack software according to spectral features which include frequency (Y-axis, kHz), time (X-axis, ms), and power intensity (color scale, dB). Calls are categorized based on frequency modulation, shape, and complexity. These spectrograms are intended as qualitative examples only. All USV call types were categorized according to Metris Sonotrack Call Classification which is based upon call classification principles described in Portfors (2007). Additionally, ‘syllables’ refer to individual vocal elements within a call and are synonymous with Sonotrack’s definition of ‘elements’ (Portfors 2007): **(A)** Short Calls are brief, simple calls with short duration <15 ms. These calls are often isolated with little to no modulation. One Syllable Calls include: **(B1)** Flat Calls are calls with a nearly constant frequency throughout. This indicates no major pitch change, producing a monotone tone. **(B2)** Up Calls where the frequency increases over time corresponding to a rising pitch. **(B3)** Down Calls where the frequency decreases over time corresponding to a falling pitch. **(B4)** Chevron Calls are V- or inverted V-shaped calls with a sharp rise and fall (or fall and rise) in frequency. They are often brief and energetic. **(B5)** U-Shape Calls are a descending then ascending smooth curve in frequency. Two Syllable Calls include: **(C1)** Trailing Calls end with a long, fading frequency tail. **(C2)** Step Down Calls have one or more abrupt drops in frequency and resemble a stair-step downward. **(C3)** Step Up Calls abruptly shift upwards in frequency like stair-steps going upward. 3–4 Syllable Calls include: **(D1)** Step Double Calls are composed of two sequential step-like changes in frequency, creating a double-stepped shape. **(D2)** Complex-3 Calls contain three distinct modulations or frequency shifts and suggest more sophisticated communication. **(D3)** Complex-4 Calls are four-part calls with varying pitch directions or shapes, reflecting more nuanced or emotionally complex vocalizations. 5+ Syllable Calls include: **(E1)** Complex-5 Calls are five-segment calls with multiple frequency modulations. They are typically rare. **(E2)** Complex-5+ Calls are calls with more than five modulated components which make them highly elaborate.

Each USV call was categorized using Metris Sonotrack software based on spectral shape, duration, and modulation characteristics. Call types include short, flat, up, down, chevron, U-shape, trailing, step down, step up, step double, complex-3, complex-4, complex-5, and complex-5+ calls, among others, as defined in Portfors (2007) [30]. Individual local elements—termed “syllables” in this study—correspond to what Metris Sonotrack refers to as “elements” [20, 30]. Sample spectrograms of call types can be found in Figure 1.

Acoustic parameters analyzed for each call from each pup included [1]: time-based features of call duration, start time, and end time [2]; frequency features of frequency start, end, minimum, maximum, and average [3]; power features of power average, power maximum, and power at frequency minimum, maximum, and average; and [4] frequency modulation features of rate of frequency change, maximum upward frequency change, and maximum downward frequency change.

Calls were additionally divided into short (<15 ms) and long (>15 ms) categories for each mouse, with separate acoustic means computed within these duration groups to minimize skew from call length distribution. In multi-syllabic calls, each syllable was independently analyzed.

To enhance translational relevance, USV parameters were interpreted in light of common human acoustic measures used to assess pathological voices: jitter, shimmer, and harmonic-to-noise ratio (HNR). Jitter is a function of frequency, or pitch, which measures frequency instability in human voices. This is most closely paralleled by our measures of frequency modulation. Shimmer is a function of amplitude, or loudness, representing amplitude variation, aligning with our analysis of power-based features. Finally, while HNR, a marker of voice clarity versus noise, is not directly measured, our classification of calls based on spectral shape and modulation complexity may reflect similar characteristics of signal periodicity and noise content. These analogues support the use of this dystonia model for probing vocal-motor deficits related to cerebellar dysfunction, particularly in disorders with early vocal involvement [31].

### Cluster analysis

USVs were grouped into temporal clusters, defined as at least 2 consecutive calls where the start of each call occurred within 0.5 s of the previous call’s end. Parameters extracted per cluster included: number of clusters per mouse pup, number of calls per cluster, mean inter-call interval, and cluster duration.

### Statistical analysis

**FIGURE 2:**
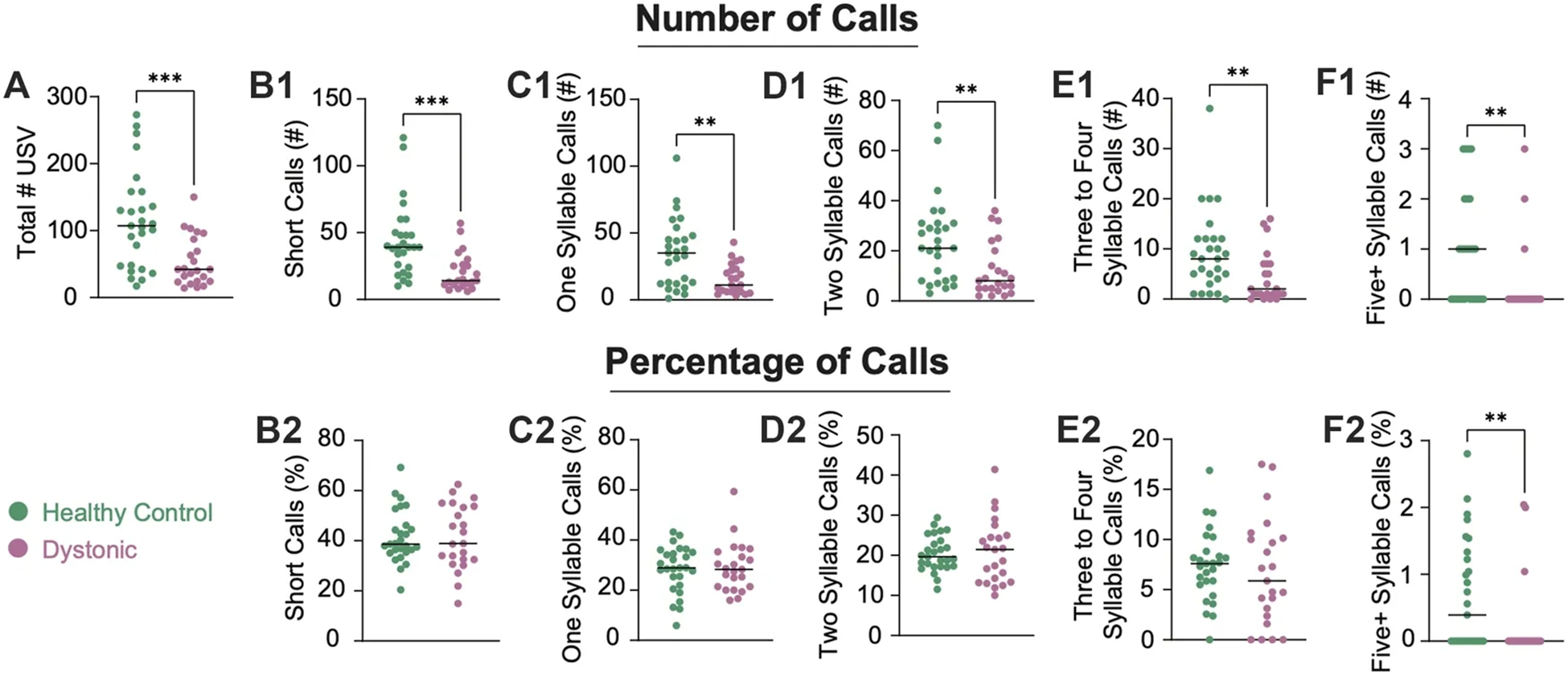
Quantitative analysis of USV call types reveals fewer total vocalizations and a statistically significant reduced percentage of the most complex calls in dystonic pups. Graphs represent individual data points and show group means for total USV count and call type distributions grouped by syllable number. Individual data points represent biological replicates (one pup; *n* = 27 healthy controls, 23 dystonic mice; experiment performed once per pup). **(A)** Total number of USVs emitted per pup. **(B1,C1,D1, E1, F1)** Number of Short, One-, Two-, Three to Four-, and Five + syllable calls emitted per pup, respectively. **(B2,C2,D2,E2,F2)** Percentage of Short, One-, Two-, Three to Four-, and Five + syllable calls emitted per pup, respectively. Syllable categorization of call types is explained in Figure 1. Statistical comparisons were performed using unpaired, two-tailed Mann-Whitney U tests. Statistically significant reductions were observed in total number of USVs, absolute numbers of all call type categories, and relative Five + syllable calls in dystonic mice compared to healthy controls. Data suggests a marked reduction in both number and complexity of vocalizations in dystonic animals. *p < 0.05; **p < 0.01; ***p < 0.001.

**FIGURE 3:**
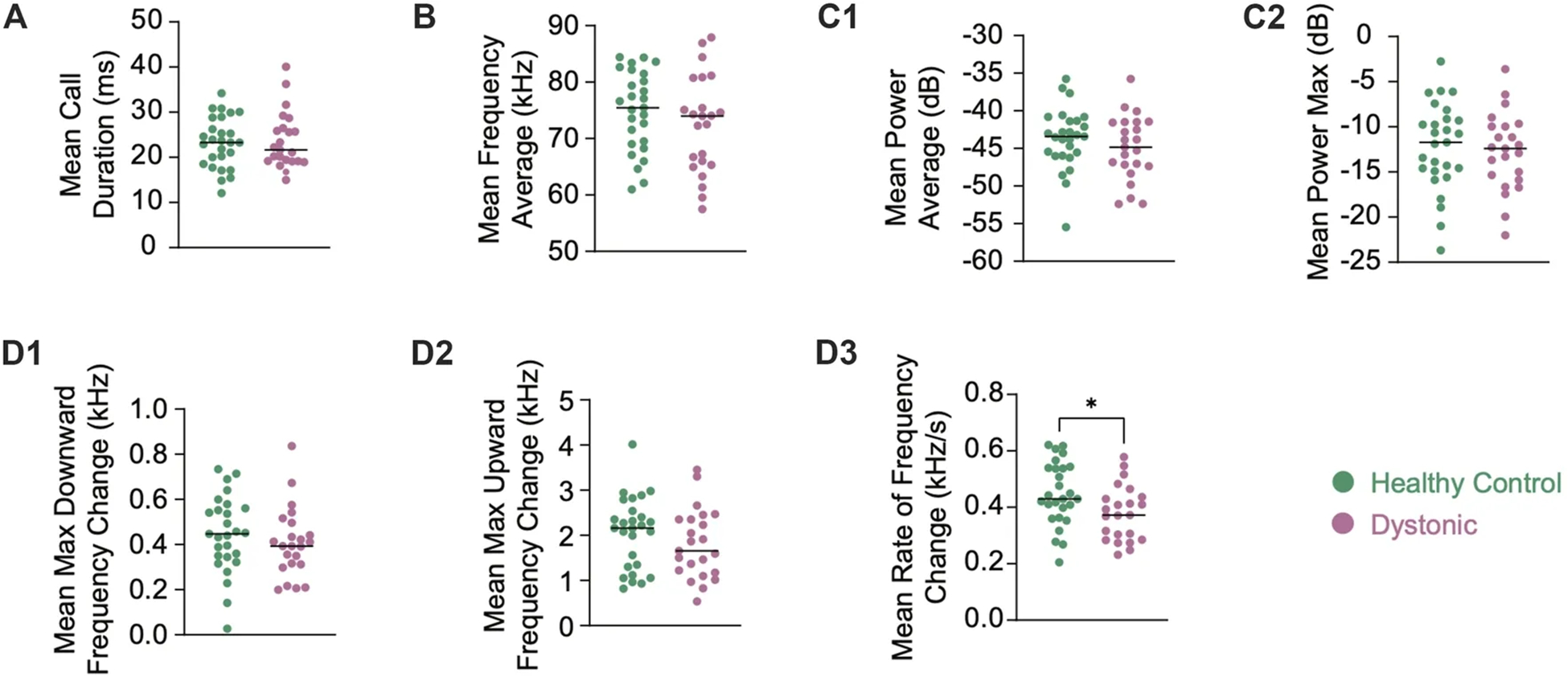
Analysis of acoustic properties of short calls indicates altered signal structure in dystonic pups. Graphs represent individual data points and show group means for various acoustic parameters of short calls (<15 ms in duration). Individual data points represent biological replicates (one pup; *n* = 27 healthy controls, 23 dystonic mice; experiment performed once per pup). **(A)** Mean short call duration (ms). **(B)** Mean frequency average (kHz). (C1,C2) Relevant power parameters of mean power average (dB) and mean power maximum (dB), respectively. **(D1–D3)** Frequency modulation parameters of mean maximum downward change in frequency (kHz), mean maximum upward change in frequency (kHz), and mean rate of frequency change (kHz s^−1^), respectively. Statistical comparisons were performed using unpaired, two-tailed Mann-Whitney U tests. Statistically significant differences were detected in upward frequency change and maximum power, indicating acoustic alterations in short calls emitted by dystonic pups. *p < 0.05; **p < 0.01; ***p < 0.001.

**FIGURE 4:**
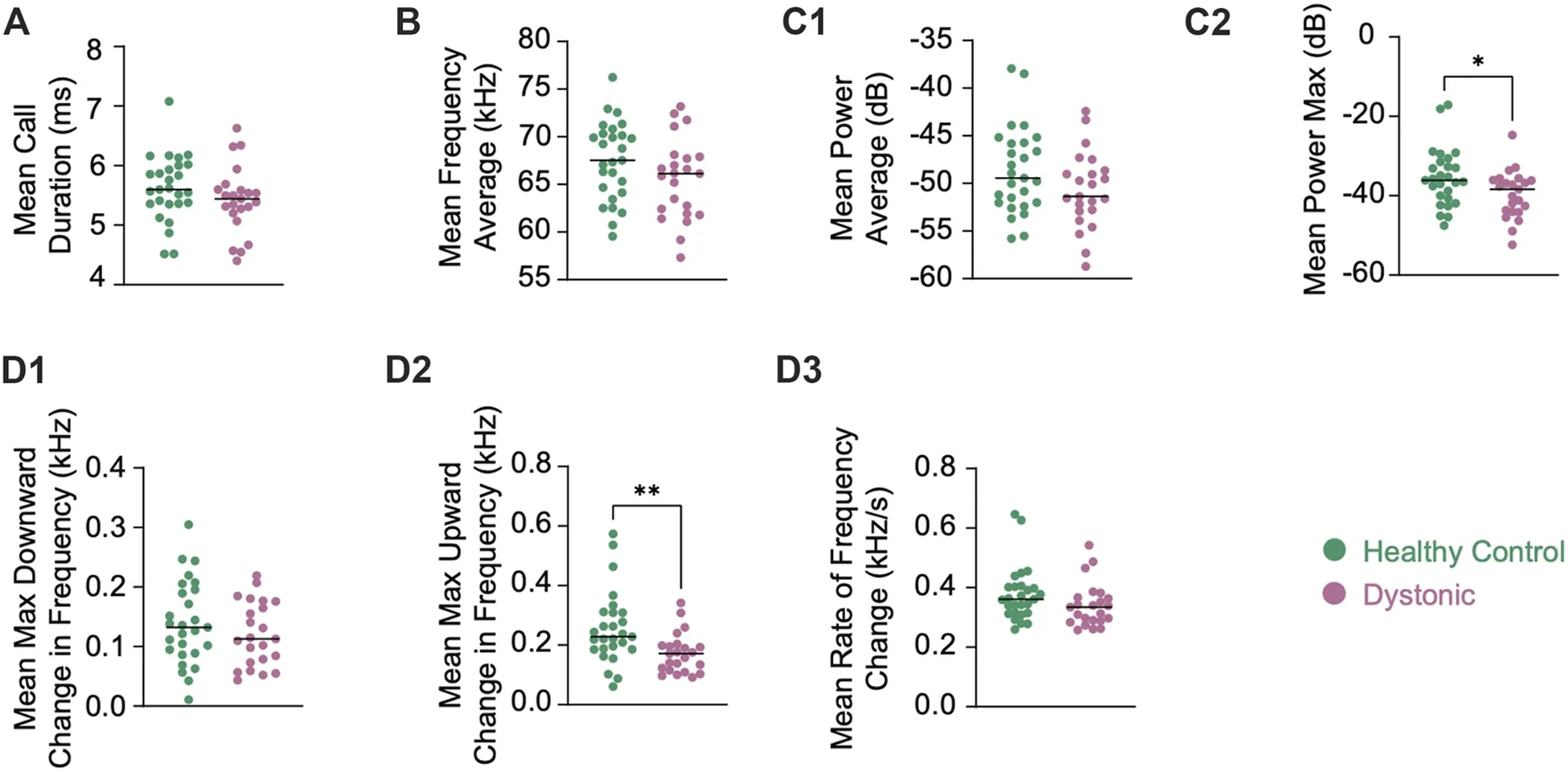
Analysis of acoustic properties of long calls shows altered signal structure in dystonic pups. Graphs represent individual data points and show group means for various acoustic parameters of long calls (>15 ms in duration). Individual data points represent biological replicates (one pup; *n* = 27 healthy controls, 23 dystonic mice; experiment performed once per pup). **(A)** Mean long call duration (ms). **(B)** Mean frequency average (kHz). (C1,C2) Relevant power parameters of mean power average (dB) and mean power maximum (dB), respectively. **(D1–D3)** Frequency modulation parameters of mean maximum downward change in frequency (kHz), mean maximum upward change in frequency (kHz), and mean rate of frequency change (kHz s^−1^), respectively. Statistical comparisons were performed using unpaired, two-tailed Mann-Whitney U tests. A statistically significant difference was detected in mean rate of frequency change, indicating acoustic alterations in long calls emitted by dystonic pups. *p < 0.05; **p < 0.01; ***p < 0.001.

**FIGURE 5:**
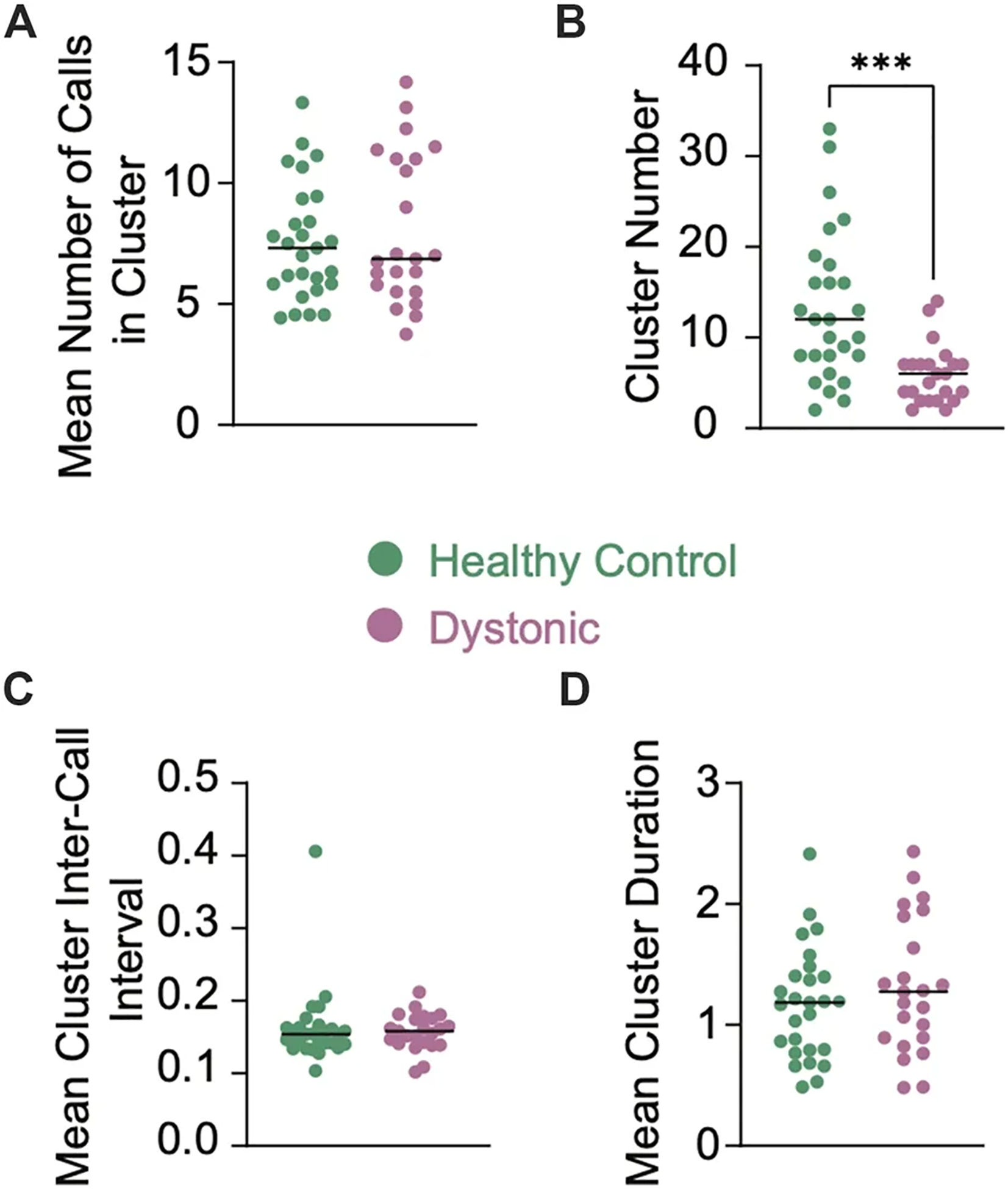
Cluster analysis of USV patterns reveals altered vocal grouping behavior in dystonic pups. Graphs represent individual data points and show group means for cluster-based metrics derived from USVs. Individual data points represent biological replicates (one pup; *n* = 27 healthy controls, 23 dystonic mice; experiment performed once per pup). A cluster was defined as a group of 2 or more unique calls where the start time of each call occurred within 0.5 s of the end time of the previous call (this also defines our inter-call interval). **(A)** Number of detected USV clusters per pup. **(B)** Mean number of calls per cluster. **(C)** Mean cluster inter-call interval (s) within clusters. **(D)** Mean cluster duration (s). Statistical comparisons were performed using unpaired, two-tailed Mann-Whitney U tests. A statistically significant reduction in the number of USV clusters in dystonic pups compared to healthy controls was observed, suggesting altered temporal organization of vocalizations. *p < 0.05; **p < 0.01; ***p < 0.001.

Averages of each acoustic parameter for each mouse were calculated in Microsoft Excel and cluster analysis parameters were extracted from the original Metris Sonotrack data set for each mouse utilizing MATLAB (Mathworks, United States). The remainder of the statistical analysis was conducted using GraphPad Prism (v10.5.0, GraphPad Software, San Diego, CA). Five more mice were excluded from the dataset after an outlier analysis. Group comparisons were performed using unpaired, two-tailed Mann-Whitney U tests due to the expected non-normal distribution of USV parameters. Spread of data was visualized via scatterplots to enhance interpretation (Figures 2–5).

Cohen’s d was used to estimate effect sizes for interpretability reported in Table 1 as group means were used for visualization and effect magnitude comparison, despite the use of nonparametric tests for significance testing, calculated using G*Power (v3.1.9.6). A post-hoc power analysis was also performed using G*Power (v3.1.9.6) for the primary endpoints of total USVs and maximum upward frequency modulation for short calls using Cohen’s d = 1.0701 and 0.8522, respectively, and samples sizes of 27 (healthy control) and 23 (dystonic mice) estimated 95% and 82%, respectively, at α = 0.05 (two-tailed), indicating adequate sensitivity. No power analysis was conducted for secondary comparisons due to the exploratory nature of these analyses.

### Data and resource availability

All raw USV data, processed statistical outputs, and custom MATLAB and Excel-based scripts for post-processing are available from the corresponding author upon reasonable request. USVs were analyzed using Metris Sonotrack (v1.4.7, Metris B.V., Netherlands).

## Results

### USV call types show quantitative reductions in cerebellar-dysfunction dystonic mice at age P9

First, to determine whether dystonic mice display altered USV patterns compared to healthy controls, we categorized calls emitted by P9 pups into call types based on shape, modulation, and syllable number using Metris Sonotrack software (Figure 1). We found that all call types were emitted by both dystonic and healthy control pups.

Total number and proportional distribution of calls across syllable-defined categories were compared between groups (Figure 2). Dystonic mice emitted statistically significantly fewer total USVs compared to healthy controls (*p* = 0.0005) (Figure 2A). Additionally, all call type categories—Short, One-, Two-, Three to Four-, and Five + syllable calls—were statistically significantly reduced in absolute number of calls in dystonic mice pups (*p* = 0.0001; *p* = 0.0020; *p* = 0.0055; *p* = 0.0068; *p* = 0.0060, respectively) (Figure 2B1,C1,D1,E1,F1). These findings suggest that cerebellar dysfunction in this dystonia model not only reduces overall vocal output but also disrupts the production of spectrally and temporally complex vocalizations, indicating impaired initiation or coordination of vocal-motor patterns early in development.

Next, we set out to investigate whether the relative proportion of call types was different between dystonic and healthy control pups. Only the percentage of the most complex vocalizations, Five + Syllable Calls, was statistically significantly reduced in dystonic mice (*p* = 0.0089) suggesting disproportionate reductions in complex call production, even if Three to Four Syllable Calls do not show a statistically significant reduction. (Figure 2E2,F2). All other percentage categories did not differ in a statistically significant manner between groups. This aligns with our hypothesis that while number of calls will uniformly be reduced in dystonic mice compared to healthy controls, the percentage of complex calls only will be reduced in dystonic mice. Ultimately, this result suggests that while overall vocal output is reduced, dystonic mice demonstrate an additional relative loss of more complex call types. This is indicative of a disrupted and/or less precise audio feedback loop given the fact that complex calls require precise audio feedback modulation. These findings confirm that core spectral features broadly are disrupted at the critical development stage and preliminarily establish this specific general dystonia mouse model’s potential for accurately capturing cerebellar vocal-motor deficits.

Additionally, as discussed in the methods, these parameters may more specifically reflect similar characteristics of signal periodicity and noise content that is typically measured by HNR in human acoustic analysis. Various human studies have shown that HNR is statistically significantly lower in patients with pathologic voices compared to controls, specifically with regard to various types of focal dysphonias which implicate specific laryngeal deficits [31, 32]. HNR has also been indicated as the most sensitive indicator of changes in the voice organ for diagnosing voice disorders in children, which the animal model at this specific timepoint studied most closely reflects [33].

### Short call acoustic parameters are altered in dystonic mice

We next examined pertinent acoustic features of Short Calls (<15 ms duration), which was consistently the predominant call category. Multiple parameters including duration, frequency, power, and frequency modulation were analyzed per pup and compared between groups (Figure 3). Based on human literature, we expected group differences between healthy control and dystonic mice to be most pronounced in frequency modulation and power as these features correspond to impaired laryngeal muscle control and audio-vocal feedback seen in vocal impairments in dystonia, with significantly less to no statistically significant differences in average vocal frequency parameters as a simple change in tonality of voice alone is not a distinct feature of laryngeal dystonia [1, 24, 31].

Compared to healthy controls, dystonic pups showed a statistically significant reduction in maximum power (*p* = 0.0347) and maximum upward change in frequency modulation (*p* = 0.0031) (Figure 3C2,D2). Other parameters reported in Figure 3 do not show statistically significant differences between the two groups. These findings indicate that dystonic mice exhibit alterations in acoustic structure, specifically in power and frequency modulation, which may parallel disruptions in human vocal control such as impaired pitch regulation and reduced intensity often seen in dystonic speech. While the mouse USV model does not measure jitter or shimmer directly, these parameters are analogous to instability in pitch and amplitude, respectively, and reductions here could reflect underlying cerebellar dysregulation in motor timing, force, and coordination, although the directionality of change differs from some human findings [31, 34].

### Long call acoustic parameters are also altered in dystonic mice but more limited

We separately analyzed Long Calls (>15 ms duration) for comparable acoustic parameters (Figure 4). Among all variables tested, only mean rate of frequency change differed between groups in a statistically significant manner (*p* = 0.0314) (Figure 4D3). Other frequency modulation, duration, frequency, and power parameters did not show statistically significant differences. While this data suggests nominal acoustic changes in longer calls, potentially indicating that more complex vocalizations may be preserved in spectral quality despite being less frequent in dystonic mice, the fact that a frequency modulation parameter did show a statistically significant reduction in dystonic mice and it is a different one than what was present in short calls leads us to reasonably come to similar conclusions as our short call results—this result is a product of reduced laryngeal muscle coordination.

### Call clustering patterns differ in dystonic mice

Finally, to investigate if and how USV patterns evolve temporally between our two groups, we analyzed call clustering behavior across pups. We defined clusters as sequences of two or more unique calls in a row in which the start time of each call was within 0.5 s of the end time of the previous call. Inter-call intervals are defined as the time between the end of a prior call and the start of the next call. This metric reflects the organization of vocal bursts which can potentially offer insight into vocal planning, effort, and audio-vocal feedback function. While average duration of clusters, average number of calls in clusters, and average cluster inter-call interval did not differ in a statistically significant way between groups (Figures 5B–D), dystonic pups did exhibit a statistically significant reduction in the total number of clusters compared to healthy controls (*p* = 0.0001) (Figure 5A). This finding suggests that while the structure of each vocal cluster may be preserved, dystonic mice produce fewer discrete bouts of vocal activity overall. This finding aligns with our finding that dystonic mice produce a statistically significant reduction in total number of USVs and seemingly simplified vocal behavior in these pups. This may reflect impairments in the initiation or coordination of vocal bursts in dystonic mice.

For all parameters discussed above with a statistically significant result, medium to large effect sizes were seen, but some parameters that did not show statistical significance showed a small effect size (Table 1). Overall, successfully identifying these vocalization abnormalities is a promising step towards validating our cerebellar dystonia mouse model as an adaptable model for studying general cerebellar contributions to vocal-motor dysfunction, including aspects relevant to childhood-onset dystonias and early disruptions in phonation. In addition to the spectral and structural call abnormalities observed, our findings that dystonic mice emitted a statistically significant fewer amount of temporal call clusters compared to healthy controls suggest that dystonic pups produce fewer calls, simpler calls, and demonstrate altered organization of vocal output over time. These findings all support the idea that cerebellar dysfunction impacts not only vocal content but also how vocalizations are sequenced and structured across time, substantiating the use of this generalized dystonia model to study cerebellar contributions to vocal-motor coordination and audio-vocal feedback. While not a direct model for laryngeal dystonia, the core features disrupted—frequency modulation, power, and call complexity—overlap with impairments observed in human vocal impairment disorders. This lays foundational groundwork for further validation studies and future research into disease mechanisms and therapeutic strategies.

## Discussion

### Cerebellar dysfunction disrupts both the complexity and timing of vocal-motor output in neonatal mice

Our findings highlight that cerebellar dysfunction alters not only the complexity but also the temporal architecture of USVs in our dystonic mouse model. These findings also align with prior work showing that the cerebellum acts as an integrative hub for temporal and spatial coordination of speech motor output [35]. Additionally, the observed statistically significant reduction in clustered vocal sequences suggest disrupted sequencing or initiation of vocal-motor bursts, supporting recent findings that implicate the cerebellum in temporal coordination and volitional control of speech in dystonia [1, 36].

Though mice do not produce human speech, their clustered USV emissions offer a behavioral analogue of structured phonation. Therefore, the general pattern of our findings in this initial study are incipiently consistent with human studies of vocal pathologies in which patients exhibit impaired audio-vocal feedback control and delayed or fragmented speech initiation and are promising results towards establishing this mouse model as a novel animal model for laryngeal dystonia [37–39]. Our cluster analysis revealed that while the structure within clusters (call number per cluster, average cluster duration, and average inter-call latency) were relatively preserved, the frequency of initiating discrete clusters was reduced in dystonic mice in a statistically significant manner. This strengthens the suggestion that cerebellar output is particularly essential for initiating and sustaining patterned vocal behaviors rather than modulating fine timing of each vocalization within a cluster.

In summary, these findings underscore the utility of this specific cerebellum-targeted generalized dystonia model in studying both spectral and temporal features of vocal-motor coordination which are also key components disrupted in human vocal deficits in dystonia. Importantly, this reinforces the need for future behavioral paradigms that test cerebellar contributions to vocalizations under stress or task-specific conditions, better approximating the demands of human speech [24].

### Establishing face validity through frequency modulation and call complexity as translational biomarkers for vocal impairments in human dystonia

The frequency modulation changes and reduction in complex call types we identified in dystonic mice resemble observations in vocal impairments in human dystonias. Patients with laryngeal dystonia often demonstrate abnormal pitch control, exaggerated pitch-shift responses, and reduced adaptive responses to altered auditory feedback [37, 40]. Recapitulating features seen in pathological voice patients, our dystonic mice showed statistically significant reductions in maximum upward frequency modulation in short calls and rate of frequency change in long calls. These parallels contribute to the relevance of our endpoints as potential behavioral biomarkers across species.

Furthermore, the observed loss of spectrally rich, complex calls in dystonic mice resemble voice breaks documented behaviorally in human disordered phonation, although direct cerebellar correlates in patient acoustic studies remain to be determined. Thus, the mouse USV system provides a simplified yet powerful platform for investigating circuit-level contributors to disordered phonation.

Although the current study does not claim to fully recapitulate the clinical features of laryngeal dystonia, the observed vocal-motor disruptions support the utility of this cerebellar dystonia model in examining early circuit-level contributions to phonation.

### Comparison with human acoustic metrics: jitter, shimmer, and harmonic-to-noise ratio

While our study focused on frequency modulation and power parameters, we sought to draw parallels to established human acoustic metrics such as jitter (cycle-to-cycle frequency instability), shimmer (amplitude instability), and harmonic-to-noise ratio (HNR), which are commonly altered in voice disorders including laryngeal dystonia. Notably, human studies have typically reported increased jitter and shimmer in affected individuals, reflecting greater instability in pitch and loudness [31, 34]. In contrast, our dystonic mice exhibited statistically significant reductions in parameters analogous to frequency modulation and power.

This apparent discrepancy may be due to several factors. First, human acoustic studies often analyze adult patients with longstanding disease, whereas our data come from P9 mice during early vocal development. One study of exploring acoustic parameters in children with voice disorders showed some variability in these patterns compared to adult studies [33]. This developmental context may reflect a different stage of circuit dysfunction characterized more by under-activation or failure to initiate complex vocal gestures than by unstable or erratic circuitry. Second, the mouse USV system, while valuable, captures a narrower range of phonatory dynamics than spoken language. Finally, our model reflects generalized cerebellar dysfunction compared to isolated laryngeal motor pathology.

Nonetheless, consistent disruptions in key acoustic parameters suggest that this model captures important aspects of cerebellar vocal-motor control. Future studies examining longitudinal vocal development and adult phenotypes will clarify whether jitter-, shimmer-, and HNR-like features evolve over time and converge more closely with human data.

### Limitations and future directions

While our findings are promising, several limitations must be acknowledged. First, USVs are an indirect proxy for phonation and, while useful, do not capture the full scope of laryngeal biomechanics. Second, the generalized dystonia model may not fully replicate the task-specific nature of human dystonias with vocal features [16]. Future experiments will potentially involve collaborations with voice centers and speech pathologists to bridge the behavioral phenotype with human vocal pathology more directly. For example, leveraging data from human laryngeal scope studies and pitch-control paradigms could strengthen translational parallels.

Our study focuses on postnatal day 9 developmentally. This is a timepoint at which pup vocalizations serve key communicative and regulatory roles. Given that human dystonias with vocal features can emerge in childhood but also adulthood, it is essential to determine whether the observed abnormalities persist in later developmental stages. A follow-up study of adult vocal behaviors will help to determine whether these early deficits persist in adulthood and provide insight into their progression and stability.

Ultimately, our cerebellum-specific conditional knockout dystonia mouse model opens the door to new opportunities for mechanistic and interventional studies for focal, vocal-specific dystonias. Additionally, vocal abnormalities in dystonia frequently emerges early in genetic forms of dystonia, such as DYT6, THAP1-associated dystonia, often preceding other motor symptoms. This early laryngeal involvement underscores its importance as a critical treatment target in genetically susceptible individuals [12–16]. Transcranial magnetic stimulation (TMS), DBS, and pharmacological approaches could be applied in this model to test circuit reversibility or compensation—strategies that are actively being explored in human trials [38]. By continuing to develop this mouse model in conjunction with human data, we move closer to a viable preclinical model for mechanistic exploration of cerebellar contributions to phonation and to identify therapeutic targets relevant to early onset dystonias and voice disorders.

## Figures and Tables

**TABLE 1:** Summary of p-values and effect sizes for all reported parameters.

Data type	Figure panel	Measurement	P-value	Effect size (d)
USV Call types	Figure 2A	Total # USV	**0.0005**	1.0701
	Figure 2B1	Short calls (#)	**0.0001**	1.085549
	Figure 2C1	One syllable calls (#)	**0.002**	0.9853495
	Figure 2D1	Two syllable calls (#)	**0.0055**	0.8326177
	Figure 2E1	Three to four syllable calls (#)	**0.0068**	0.7467018
	Figure 2F1	Five + syllable calls (#)	**0.006**	0.7027988
	Figure 2B2	Short calls (%)	0.9654	0.01529402
	Figure 2C2	One syllable calls (%)	0.8431	0.09372114
	Figure 2D2	Two syllable calls (%)	0.8508	0.08527367
	Figure 2E1	Three to four syllable calls (%)	0.4597	0.1449143
	Figure 2F2	Five + syllable calls (%)	**0.0089**	0.6574987
Properties of short calls	Figure 3A	Mean Call duration (ms)	0.1468	0.3598355
	Figure 3B	Mean frequency average (kHz)	0.111	0.4752404
	Figure 3C1	Mean power average (dB)	0.1509	0.4801589
	Figure 3C2	Mean power max (dB)	**0.0347**	0.6327602
	Figure 3D1	Mean max downward change in frequency (kHz)	0.3533	0.29954
	Figure 3D2	Mean max upward change in frequency (kHz)	**0.0031**	0.8522158
	Figure 3D3	Mean rate of frequency change (kHz/s)	0.0731	0.4368165
Properties of long calls	Figure 4A	Mean Call duration (ms)	0.9385	0.06397373
	Figure 4B	Mean frequency average (kHz)	0.2533	0.3083129
	Figure 4C1	Mean power average (dB)	0.2784	0.2968299
	Figure 4C2	Mean power max (dB)	0.5114	0.1302836
	Figure 4D1	Mean max downward change in frequency (kHz)	0.2374	0.2245653
	Figure 4D2	Mean max upward change in frequency (kHz)	0.3239	0.3198195
	Figure 4D3	Mean rate of frequency change (kHz/s)	**0.0314**	0.6598893
Cluster analysis of USV patterns	Figure 5A	Mean number of calls in cluster	0.7465	0.1853972
	Figure 5B	Cluster number	**0.0001**	1.161637
	Figure 5C	Mean cluster inter-call interval (s)	0.5527	0.1323731
	Figure 5D	Mean cluster duration (s)	0.4141	0.2859509

For effect size; small: d = 0.2–0.5; medium: d = 0.5–0.8; large: d > 0.8.

Bolded values in P-value column are statistically significant.

## Data Availability

The raw data supporting the conclusions of this article will be made available by the authors, without undue reservation.

## References

[1] Kshatriya N, Battistella G, Simonyan K. Structural and functional brain alterations in laryngeal dystonia: a coordinate-based activation likelihood estimation meta-analysis. Hum Brain Mapp (2024) 45:e70000. doi:10.1002/hbm.70000 39305101 PMC11415616

[2] Simonyan K, Ludlow CL. Abnormal activation of the primary somatosensory cortex in Spasmodic dysphonia: an fMRI study. Cereb Cortex (2010) 20:2749–59. doi:10.1093/cercor/bhq023 20194686 PMC2951850

[3] Dwenger K, Roy N, Jennings SG, Smith ME, Mathy P, Simonyan K, Comparing the effects of sensory tricks on voice symptoms in patients with laryngeal dystonia and essential vocal tremor. Lang Hearing Res (2025) 68: 1654–75. doi:10.1044/2024_JSLHR-24-00476 PMC12381841 40014398

[4] Edgar JD, Sapienza CM, Bidus K, Ludlow CL. Acoustic measures of symptoms in abductor Spasmodic Dysphonia. J Voice (2001) 15:362–72. doi:10.1016/S0892-1997(01)00038-8 11575633

[5] Kim JH, Larson CR. Modulation of auditory-vocal feedback control due to planned changes in voice f_o_. J Acoust Soc Am (2019) 145:1482. doi:10.1121/1.5094414 31067945 PMC6433561

[6] Larson CR, Robin DA. Sensory processing: advances in understanding structure and function of pitch-shifted auditory feedback in voice control. AIMS Neurosci (2016) 3:22–39. doi:10.3934/Neuroscience.2016.1.22

[7] Ludlow CL, Domangue R, Sharma D, Jinnah HA, Perlmutter JS, Berke G, Consensus-based attributes for identifying patients with Spasmodic dysphonia and other voice disorders. JAMA Otolaryngol Head Neck Surg (2018) 144:657–65. doi:10.1001/jamaoto.2018.0644 29931028 PMC6143004

[8] Sapienza CM, Walton S, Murry T. Acoustic variations in adductor Spasmodic dysphonia as a function of speech task. J Speech Lang Hear Res (1999) 42:127–40. doi:10.1044/jslhr.4201.127 10025549

[9] Radmard S, Zesiewicz TA, Kuo S. Evaluation of cerebellar ataxis patients. Neur Clin (2023) 41:21–44. doi:10.1016/j.ncl.2022.05.002 PMC10354692 36400556

[10] Liu Y, Chen F, Liang F, Wang C, Chen D, Zhou J, Comparison of the efficacy and adverse effects of unilateral or bilateral botulinum toxin injections for adductor Spasmodic dysphonia: a systematic review and meta-analysis. Eur Arch Otorhinolaryngol (2024) 281:1357–69. doi:10.1007/s00405-023-08366-2 38095707 PMC10858140

[11] Benninger MS, Smith LJ. Noncosmetic uses of botulinum toxin in otolaryngology. Clev Clin J Med (2015) 82:729–32. doi:10.3949/ccjm.82a.14096 26540323

[12] Artusi CA, Dwivedi A, Romagnolo A, Bortolani S, Marselli L, Imbalzano G, Differential response to pallidal deep brain stimulation among monogenic dystonias: systematic review and meta-analysis. J Neurol Neurosurg. Psychiatry (2020) 91:426–33. doi:10.1136/jnnp-2019-322169 32079672

[13] Kupsch A, Benecke R, Müller J, Trottenberg T, Schneider G, Poewe W, Pallidal deep-brain stimulation in primary generalized or segmental dystonia. New Eng J Med (2006) 355:1978–90. doi:10.1056/NEJMoa063618 17093249

[14] Bressman SB, Sabatti C, Raymond D, de Leon D, Klein C, Kramer PL, The DYT1 phenotype and guidelines for diagnostic testing. Neurology (2000) 54: 1746–52. doi:10.1212/wnl.54.9.1746 10802779

[15] Ozelius L, Lubarr N. DYT1 early-onset isolated dystonia. DYT-TOR1A. In Adam MP, Bick S, Mirzaa GM, editor. GeneReviews^®^ [Internet]. Seattle (WA): University of Washington, Seattle (1999). 1993–2026. Available online at: https://www.ncbi.nlm.nih.gov/books/NBK1492/# (Accessed June 20, 2025).

[16] Djarmati A, Schneider SA, Lohmann K, Winkler S, Pawlack H, Hagenah J, Mutations in *THAP1* (*DYT6*) and generalised dystonia with prominent Spasmodic dysphonia: a genetic screening study. The Lancet Neurol (2009) 8: 447–52. doi:10.1016/S1474-4422(09)70083-3 19345148

[17] Roy N, Awan SN, Jennings S, Jensen J, Merrill RM (2024). Adductor laryngeal dystonia *versus* muscle tension dysphonia: examining the utility of automated acoustic analysis to detect task dependency as a distinguishing feature, J Speech Lang Hear Res. 67, 3612–30. doi:10.1044/2024_JSLHR-24-00104 39259876

[18] Arriaga G, Jarvis ED. Mouse vocal communication system: are ultrasounds learned or innate? Brain Lang (2013) 124:96–116. doi:10.1016/j.bandl.2012.10.002 23295209 PMC3886250

[19] Rey Hipolito AG, van der Heijden ME, Sillitoe RV. Chapter six -Physiology of dystonia: animal studies. Int Rev. Neurobiol (2023) 169:163–215. doi:10.1016/bs.irn.2023.05.004 37482392

[20] Lahvis GP, Alleva E, Scattoni ML. Translating mouse vocalizations: prosody and frequency modulation. Genes Brain Behav (2011) 10:4–16. doi:10.1111/j.1601-183X.2010.00603.x 20497235 PMC2936813

[21] Radic R, Lukacova K, Baciak L, Hodova V, Kubikova L. The role of cerebellum in learned vocal communication in adult songbirds. Sci Rep (2024) 14:8168. doi:10.1038/s41598-024-58569-8 38589482 PMC11001874

[22] Ursu R, Centeno E, Leblois A. A lobule-specific neuronal representation of song temporal structure in the songbird cerebellum. eLife (2026). 15:RP109802. doi:10.7554/eLife.109802.1

[23] Brandenburg C, Srivastava S, Rey Hipolito AG, Lin T, Arenkiel BR, Sillitoe RV. Frequency-dependent cerebellar circuits independently gate social vocalizations and movement. Preprint on Biorxiv (2026):2026.02.18.706564. doi:10.64898/2026.02.18.706564

[24] Marchese MR, Longobardi Y, Libero R, Yesilli-Puzella G, D’Alatri L, Galli J. Lombard effect and voice changes in adductor laryngeal dystonia: a pilot study. Laryngoscope (2024) 134:3754–60. doi:10.1002/lary.31491 38727193

[25] Brown AM, van der Heijden ME, Jinnah HA, Sillitoe RV. Cerebellar dysfunction as a source of dystonic phenotypes in mice. Cerebellum (2023) 22: 719–29. doi:10.1007/s12311-022-01441-0 35821365 PMC10307717

[26] White JJ, Sillitoe RV. Genetic silencing of olivocerebellar synapses causes dystonia-like behaviour in mice. Nat Commun (2017) 8:14912. doi:10.1038/ncomms14912 28374839 PMC5382291

[27] Altman J, Bayer SA. Development of the cerebellar system: in relation to its evolution, structure, and functions. Boca Raton, USA: CRC Press (1997).

[28] Yin X, Chen L, Xia Y, Cheng Q, Yuan J, Yang Y, Maternal deprivation influences pup ultrasonic vocalizations of C57BL/6J mice. PLoS ONE (2016) 11: e0160409. doi:10.1371/journal.pone.0160409 27552099 PMC4994965

[29] van der Heijden ME, Gill JS, Rey Hipolito AG, Salazar Leon LE, Sillitoe RV. Quantification of behavioral deficits in developing mice with dystonic behaviors. Dystonia (2022) 1:10494. doi:10.3389/dyst.2022.10494 36960404 PMC10032351

[30] Portfors CV. Types and functions of ultrasonic vocalizations in laboratory rats and mice. J Am Assoc Lab Anim Sci (2007) 46:28–34. 17203913

[31] Teixeira JP, Fernandes PO. Acoustic analysis of vocal dysphonia. Proced Comp. Sci (2015) 64:466–73. doi:10.1016/j.procs.2015.08.544

[32] Teixeira JP, Oliviera C, Lopes C. Vocal acoustic analysis—jitter, shimmer and HNR parameters. Proced Tech (2013) 9:1112–22. doi:10.1016/j.protcy.2013.12.124

[33] Niedzielska G Acoustic analysis in the diagnosis of voice disorders in children. Int J Ped Otorhinolaryngol (2001) 57:189–93. doi:10.1016/S0165-5876(00)00411-0 11223450

[34] Carrillo L, Ortiz KZ. Vocal analysis (auditory and acoustic) of dysarthrias. Pró-fono R Atual Cient (2007) 19:381–6. doi:10.1590/S0104-56872007000400010 18200388

[35] Simonyan K, Fuertinger S. Speech networks at rest and in action: interactions between functional brain networks controlling speech production. J Neurophysiol (2015) 113:2967–78. doi:10.1152/jn.00964.2014 25673742 PMC4416556

[36] Kothare H, Schneider S, Mizuiri D, Hinkley L, Bhutada A, Ranasinghe K, Temporal specificity of abnormal neural oscillations during phonatory events in laryngeal dystonia. Brain Comms (2022) 4:fcac031. doi:10.1093/braincomms/fcac031 PMC8962453 35356032

[37] Behroozmand R, Sangtian S. Neural bases of sensorimotor adaptation in the vocal motor system. Exp Brain Res (2018) 236:1881–95. doi:10.1007/s00221-018-5272-9 29696312

[38] Rogić Vidaković M, Šoda J, Kuluva JE, Bošković B, Dolić K, Gunjača I. Exploring neurophysiological mechanisms and treatment efficacies in laryngeal dystonia: a transcranial magnetic stimulation approach. Brain Sci (2023) 13:1591. doi:10.3390/brainsci13111591 38002550 PMC10669610

[39] Weerathunge HR, Tomassi NE, Stepp CE. What can altered auditory feedback paradigms tell us about vocal motor control in individuals with voice disorders? Perspect ASHA Spec Interest Groups (2022) 7:959–76. doi:10.1044/2022_PERSP-21-0019 37397620 PMC10312128

[40] Thomas A, Mirza N, Eliades SJ. Auditory feedback control of vocal pitch in Spasmodic dysphonia. The Laryngoscope (2021) 131:2070–5. doi:10.1002/lary.29254 33169850

[41] Fitzgerald AF, Coello JA, Lyon AM, Dao BL, van der Heijden ME. Modeling laryngeal dystonia through spectral analyses of vocalizations in a dystonia mouse model. bioRxiv (2025):2025.07.07.663183. doi:10.1101/2025.07.07.663183 PMC13256318 42294485

